# Mating-induced sexual inhibition in the jumping spider *Servaea incana* (Araneae: Salticidae): A fast-acting and long-lasting effect

**DOI:** 10.1371/journal.pone.0184940

**Published:** 2017-10-18

**Authors:** Vivian Mendez, Rowan H. McGinley, Phillip W. Taylor

**Affiliations:** Department of Biological Sciences, Macquarie University, Sydney, Australia; Scientific Research Centre of the Slovenian Academy of Sciences and Art, SLOVENIA

## Abstract

Mating-induced sexual inhibition has been studied extensively as an important facet of many insect mating systems but remains little understood in spiders. Once mated, females of many spider species become unreceptive and aggressive toward males, but the speed of onset and persistence of this effect are not known. Addressing this gap, the present study considers (1) mating tendency of virgins, latency to remating, and lifetime mating frequency and (2) how quickly sexual inhibition is expressed after the first mating in female *Servaea incana* jumping spiders. Encounters between males and females took place in two contexts that simulated locations where mating occurs in nature: in the light away from nests (‘in the open’) and in low light within the shelter of silken retreats (‘at a retreat’). Virgin females exhibited high receptivity levels in both contexts but sexual inhibition was induced immediately after their first copulation. The most common tendency was for just one mating in a lifetime, and few females mated more than twice. Context also had an effect on female mating tendency, as virgin females in the open rejected more males before accepting their first mate than did virgin females in retreats. Considering only those females that did remate, females in the open tended to reject fewer males before remating. Given low levels of female remating, virgin females appear to be at a premium for male reproductive fitness in *S*. *incana* jumping spiders and this is a likely explanation for protandry found in nature.

## Introduction

Female reproductive decisions are key to fitness of both sexes. For females, mating decisions are important for the acquisition of sperm to fertilize eggs, and sometimes also for the acquisition of resources to sustain themselves and their offspring (Thornhill and Alcock, [1,2,3]. Females tend to be selective about which males they will accept as a mate and, as a consequence, preferred male types accrue a disproportionate share and there tends to be substantial variation in male reproductive success [4,5,6]. In some cases, variation in male fitness is driven not only by female mate preferences but is also driven by female post-copulatory decisions of whether or when to accept a subsequent suitor [7,8,9].

Females of many species are able to store sperm, and a male’s sperm may be displaced or diluted by those of the female’s subsequent mates [10]. The presence of ejaculates from multiple males leads to sperm competition, whereby the sperm from two or more males compete for the fertilization of ova [11,12]. Given that male fitness can be severely reduced by competing ejaculates, there is selection both for increased competitive ability of ejaculates and also for minimizing the risk of sperm competition. For example, males might physically defend their mates against rival males to reduce the risk of sperm competition [13,14,15] or may deposit a mating plug that impedes insemination by rivals [16,17,18,19,20]. Alternatively, a male’s ejaculate can be protected from sperm competition in his absence if females are unwilling to accept subsequent suitors through mating-induced sexual inhibition.

Mating-induced sexual inhibition has been reported for numerous insects [for reviews, see 21,7,22,23] and has been studied in detail in several insect systems, including drosophilid flies [24,25,26], tephritid flies [27,28,29] and bruchid beetles [30,31]. While the expression and mechanisms are far less known, mating-induced sexual inhibition has also been reported in arachnids, including ticks [31,32], and spiders of the families Agelenidae [33,34,17], Linyphiidae [35,36,37], Salticidae [38,39,40], Lycosidae [41,42,43], Theridiidae [44,45], Tetragnathidae [46,47], Pholcidae [48,49], Eresidae [50], and Thomisidae [51]. Although reports of mating-induced sexual inhibition in spiders span a broad taxonomic range, most studies have done little more than record the incidence of this phenomenon at the population level, and usually only over a few days following the female’s first mating. To understand the evolutionary significance of mating-induced sexual inhibition in spiders it is important that effects be considered over the full female lifespan.

Jumping spiders (Araneae: Salticidae) are among the most studied spiders in terms of mating strategies and precopulatory courtship behaviours [52,53,54,55,56]. Jumping spiders have ‘primary’ eyes that provide them acute vision [57,58], as well as an array of ‘secondary’ eyes that serve as motion detectors [59,60,61] and also provide low acuity vision [62]. Rather than building webs, most jumping spiders are cursorial hunters that use their remarkable vision to navigate [63] as well as to locate, identify, and pursue prey [64,65,66,67]. In accord with their visual abilities and active lifestyle, jumping spiders also rely on vision for communication and are best known for their elaborate courtship routines [68,69,70,71]. Jumping spider courtship routines typically entail complex postures and motions of legs, pedipalps and soma, as the males ‘dance’ and pursue a prospective mate and, if successful, mate in the open [72,73]. While jumping spiders are best known for their vision-mediated courtship routines, they are also known for their use of seismic and tactile signals to court females and then mate in sheltered locations or silken retreats where visual signals would be occluded by darkness or sheets of silk [74]. Given the marked differences in communication and assessment environment of these mating contexts [75,76], there is a need to consider potential differences in efficacy of mating interactions both in terms of mating tendency and in terms of subsequent female mating decisions. In some jumping spiders, matings that take place in sheltered locations last longer than those that take place in the open [38,39], and the effects of such differences in mating behaviour on induction of mating-induced sexual inhibition also warrant consideration.

Several studies have documented mating-induced sexual inhibition in jumping spiders. Jackson [77] found that virgin females of *Phidippus johnsoni* were more willing to copulate than were mated females. Mating tendency can also vary with context. For example, virgin females of *Trite planiceps* are more likely to mate, and to later remate, if in the shelter of a rolled-up leaf rather than in the open [39]. Similarly, females of *P*. *johnsoni* [38] and *Myrmarachne lupata* [78] exhibit higher receptivity when in sheltered locations of silken retreats. Copulations in the open are thought to expose females to greater risk of predation than copulations in sheltered locations, and females appear to reduce this risk through decreased mating tendency.

As is the case for other spider taxa, reports of mating-induced sexual inhibition in jumping spiders have only included observations made during the first days or at most weeks following a female’s first mating. Given that female jumping spiders might store and use sperm from their first mate throughout their remaining lifetime [38], to understand the evolutionary significance of mating-induced sexual inhibition in these spiders there is a need for studies that consider the persistence and efficacy of sexual inhibition over this time frame. Male jumping spiders typically decamp immediately after copulation ends but there are no studies of how quickly sexual inhibition is induced in females. In the present study we address both of these issues through studies of *Servaea incana* (Karsch, 1878) (= *Servaea vestita* (L. Koch, 1879)), a common jumping spider in temperate regions of Australia [79].

*Servaea incana* is commonly associated with eucalyptus trees, where females build silken retreats and nests in dry cavities that form under loose bark. Retreats are thin structures built as shelter whereas nests are denser and built as shelter for both adult females and their eggs; for the purposes of this study, both are referred to as ‘retreats’. *Servaea incana* males use visual displays to court females that they encounter in the open or at retreats, and may also use seismic courtship at retreats. Unreceptive females usually fend off the male with their legs and turn away. Receptive females usually remain still and lower their body to the substrate, allowing the male to mount [80]. In the present study, for courtship and mating that takes place in the open and at retreats we investigate (1) female mating tendency, (2) mating duration, (3) presence and persistence of mating-induced sexual inhibition in females following their first mating, and (4) how quickly mating-induced sexual inhibition is expressed.

## Materials and methods

### Spider origin and maintenance

Adult males, immature males, and immature females of *S*. *incana* were collected from parks in Sydney, Australia, between December 2010 and September 2011. Permissions were not required for collecting at the parks, as *S*. *incana* is not an endangered or protected invertebrate. Spiders were housed individually in a controlled environment laboratory (25±0.5°C, 65±5% RH). Lighting was a mix of metal halide and halogen. The halogen lights were set to gradually ramp up to full output over a 0.5 h dawn phase, remain on full for 12 h and then ramp down again over a 0.5 h dusk phase (0.5 Dawn: 12 Light: 0.5 Dusk: 11 Dark). The metal halide lights were set to 12 Light: 12 Dark to coincide with the full illumination phase of the halogen lights. Spiders were housed in 1.125-L plastic cages of roughly cubic shape with a mesh-covered 10 cm diameter opening on one side for ventilation. As a source of water, a 5-mL vial of water was placed in each cage. A cotton dental roll inserted through a hole in the vial lid served as a wick that carried moisture into the cage. Jumping spiders can suffer reduced performance when maintained in simple laboratory cages, and this can be ameliorated by the inclusion of architecturally complex structure [81]. As environmental enrichment, each cage was loosely filled with crumpled paper. Spiders were fed two fly pupae each week, alternating between Queensland fruit fly (*Bactrocera tryoni*) and housefly (*Musca domestica*). Every two weeks, spiders were transferred to clean cages, fresh paper was provided, and the vials of water and cotton wicks were replaced. Immature males (evident from enlarged terminal segments of the pedipalps) were reared through to adulthood and were then used in mating experiments along with the males that had been collected as adults.

Sex of immature *S*. *incana* females is readily apparent once in their penultimate instar (‘subadult’ stage) from the developing epigynum visible through the cuticle. At this stage, each immature female was assigned to one of two groups that would later be used in experiments that simulate encounters between adult males and females taking place in the light away from nests (‘in the open’) and encounters taking place in low light within the shelter of silken retreats that *S*. *incana* females construct under loose bark (‘at the retreat’). *Servaea incana* males are able to mate repeatedly and were reused in this experiment with the restriction that a male that copulated with a female during a trial was not used in another trial for at least two weeks. *Servaea incana* females to be tested in the open remained in 1.125-L maintenance cages. *Servaea incana* females to be tested at the retreat were transferred to 150 mm diameter clear plastic Petri dishes. To simulate the typical site of retreats in nature, each Petri dish contained a shelter comprising a 50 x 40 mm sheet of brown paper folded to the shape of a tent. As a source of moisture, each Petri dish contained a 5-mL vial of water with a cotton wick. Spiders always moulted inside silken retreats that they constructed under the paper shelter. In nature, retreat sites may be occupied for weeks or months, leading to accumulation of silk and chemical cues. To preserve these cues, spiders maintained for tests at the retreat were not transferred to clean Petri dishes during the experimental period, but the remains of dead flies and excess silk were removed and the vials of water and cotton wicks were replaced every two weeks.

### Experiment 1: Lifetime mating tendency

The aim of this experiment was to determine for each context (1) female mating tendency, (2) mating duration, (3) induction of sexual inhibition in females following their first mating, and (4) lifetime mating frequency. To ensure that all adult females used in these experiments were virgin and of known age for the first mating opportunity, subadult females maintained in 1.125-L cages (for trials ‘in the open’) and in Petri dishes (for trials ‘at the retreat’) were monitored daily until they moulted to maturity. Virgin females participated in their first mating trial on the day after they moulted to maturity. Each adult female was exposed to a different male every day for the first ten days of adult life, and then every ten days for the rest of their lives.

Trials of matings in the open were run in a circular white arena. A clear acrylic cylinder (150 mm diameter, 120 mm high) was positioned over a 180 mm diameter disk of white plastic that was mounted on a tripod. So that spiders were not distracted by movement in the surroundings, the outside surface of the cylinder wall was covered with white paper, leaving a 10 mm gap at the bottom for video recording of interactions at floor level (Sony HDC HS700 High Definition camcorder with supplementary close-up lenses). An opaque white plastic barrier was positioned across the middle of the arena, dividing the arena into halves. A male *S*. *incana* was guided gently out of its cage with a soft paintbrush and released on one side of the barrier. A female was released on the other side of the barrier. The spiders were left in the arena for three minutes to settle down before the trial was started by removing the barrier. After the spiders oriented toward each other and interacted, if the pair did not mate the trial ended when the female ran away from the male and climbed the wall of the arena and the male failed to follow. Failure to pursue a decamping female would presumably curtail interaction in nature. If the pair mated, the trial ended when the male dismounted and the pair separated. Between trials, the arena was wiped clean with water-moistened paper towel to remove silk and pheromones [82]. Fifty-four *S*. *incana* females were tested in this context.

Trials of matings at the retreat were run in the Petri dishes containing a female in her silken retreat, constructed under the provided paper shelter. Trials started when a male was gently coaxed out of its cage with a soft paintbrush and released into the Petri dish, and were video recorded in high definition for later analysis (Sony HDC HS700). After the spiders oriented toward each other and interacted, if the pair did not mate within one hour the trial ended and the male was removed. Trials also ended if the male or the female left the arena within an hour of the start of the trial. If the pair mated then the entire copulation was video recorded through the opening of the shelter and the trial ended when the male dismounted and decamped from the female’s retreat. Thirty-five *S*. *incana* females were tested in this context.

After they died, tested spiders were preserved in 70% ethanol. After preservation spiders were photographed using a ProgResC10 digital camera (Jenoptik LOS GmbH, Germany), focussed through an Olympus SZX12 dissecting microscope (Olympus Corporation, Tokyo, Japan). Cephalothorax width, a common metric of spider size [83,84,85], was measured from digital images using ImageJ 1.36b (National Institute of Health, Bethesda, MD, USA).

### Experiment 2: Timing of sexual inhibition onset

Having established the lifetime effects of mating-induced sexual inhibition in Experiment 1, in Experiment 2 we investigated how quickly sexual inhibition is induced in *S*. *incana*. As in Experiment 1, trials were carried out in the open and at retreats using virgin females that moulted to maturity the day before testing. All trials were video recorded, using a high definition video camera (Sony HDC HS700).

Forty-four females were used in this experiment, 22 in the open and 22 at the retreat. Each virgin female was sequentially given the opportunity to mate with three males and only females that mated in the first trial were used in subsequent trials. The general approach for this experiment was the same as for Experiment 1 (see above for details). Immediately after the first mating finished, the male was removed. For trials in the open, the arena was wiped clean with water-moistened paper towel to remove silk and pheromones [82]. For trials run at the retreat, the Petri dish was wiped clean but cues inside the retreat remained.

Immediately after cleaning the arena a second male was released with each female. There was a latency of approximately five minutes between the end of the first trial and the start of the second trial. After the spiders oriented toward each other and interacted, if the pair did not mate within one hour the trial ended and the male was removed. If the pair mated, the trial ended when the male dismounted and the pair separated. Males were then removed from the arena. Three hours after the end of the second trial, the Petri dish was wiped clean and each female was provided a third mating opportunity. Females stayed in the arena continuously from the beginning of the first trial until the end of the second trial. At the end of the second trial, females used in trials in the open were returned to their cages until the start of the third trial, while females used in trials at the retreat stayed in the Petri dish that contained their retreat.

### Statistical analysis

Ordinal logistic regression was used to test the effects of mating context, female size and first mate size on number of males rejected before accepting a first mating (0, 1, 2+) and number of rematings over a lifetime (0, 1, 2+). Nominal logistic regression was used to test if tendency to remate at least once over a lifetime varied with context, the size of the female or size of the female’s first mate in either context. Ordinary least squares models were used to test the effects of mating context, female size and first mate size on duration of mounting during the first mating and, for those females that did remate, the number of males rejected before accepting a second mate. To test the possibility that effects of male or female size varied with mating context, interactions between male and female size and context were tested in all initial models although only significant interactions were retained in final models. Fisher’s exact test was used to compare contexts with respect to the number of females that mated with the first male they encountered, the number of females that mated with one of the two first males they encountered and the number of females that mated when provided two further opportunities on the day of their first mating.

## Results

### Experiment 1: Lifetime mating tendency

#### First mating

**Fig 1:**
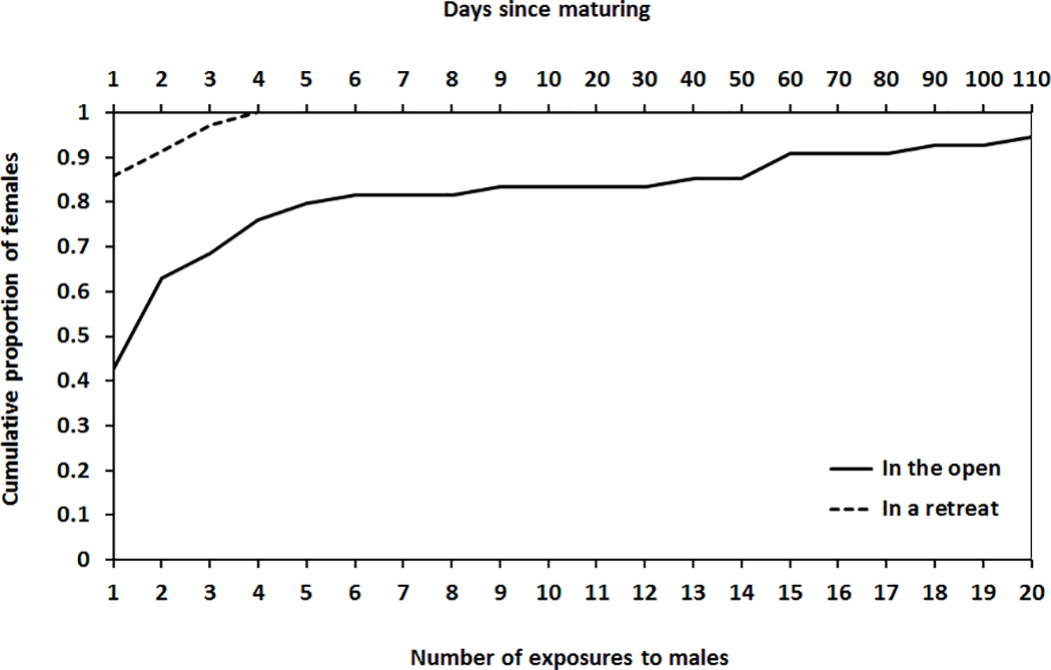
Cumulative proportion of virgin female *Servaea incana* that mated following repeated exposure to males (a) in the open (*N* = 54) or (b) in a retreat (*N* = 35).

Of the 54 females tested in the open and 35 females tested at the retreat, all except three females mated at least once (Fig 1). All of the females that did not mate had been tested in the open, and had been exposed to 3, 11, and 14 males before they died.

In trials carried out in the open 42% of females mated with the first male they encountered whereas in trials carried out at retreats 85% of females mated with the first male they encountered (Fisher’s exact test, *P* < 0.0001) (Fig 1). In trials carried out in the open 66% of females mated with one of the first two males they encountered whereas in trials carried out at retreats 91% of females mated with one of the first two males they encountered (Fisher’s exact test, *P* < 0.005). The remaining virgin females tested in the retreat (8%) mated with the third or the fourth male they encountered whereas the remaining virgin females tested in the open (31%) encountered a greater number of males (up to 20) before accepting their first mate.

The number of males rejected by virgin females before mating was greater in the open than at the retreat and varied with female size in a context-specific manner (Mating context *G*_1_ = 7.600, *P* = 0.006 [Fig 1], Female size *G*_1_ = 0.213, *P* = 0.645, Mating context x Female size *G*_1_ = 4.814, *P* = 0.028). For trials carried out in the open, larger females rejected fewer males before mating than was the case for smaller females (*G*_1_ = 7.007, *P* = 0.008) but for trials carried out at the retreat there was no evidence of relationship between female size and number of males rejected before first mating (*G*_1_ = 0.992, *P* = 0.319).

Males remained mounted for a shorter duration when mating with virgin females in the open (Least squares mean ± se = 1609 ± 204 s, range 144–4891) than when in a retreat (Least squares mean ± se = 2652 ± 248 s, range 1078–8295) (*F*_1,62_ = 9.854, *P* = 0.003) but duration of mounting was not related to either female size (*F*_1,62_ = 0.839, *P* = 0.363) or male size (*F*_1,62_ = 0.071, *P* = 0.790).

#### Remating

**Fig 2:**
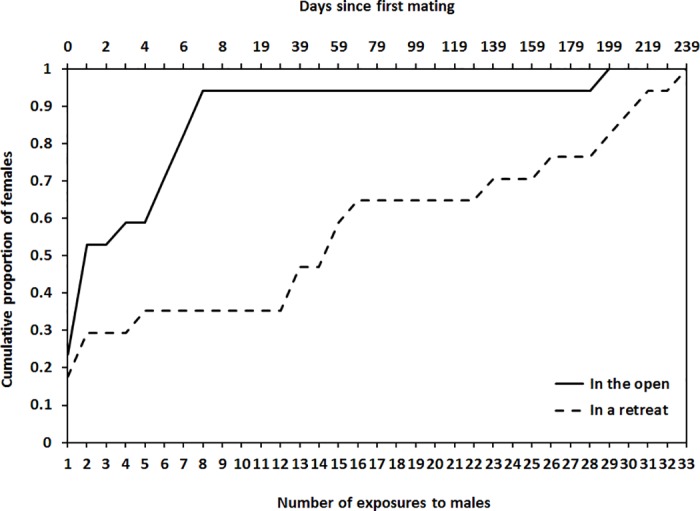
Cumulative proportion of once-mated female *Servaea incana* that remated following repeated exposure to males (a) in the open (*N* = 17) or (b) in a retreat (*N* = 17).

**Fig 3:**
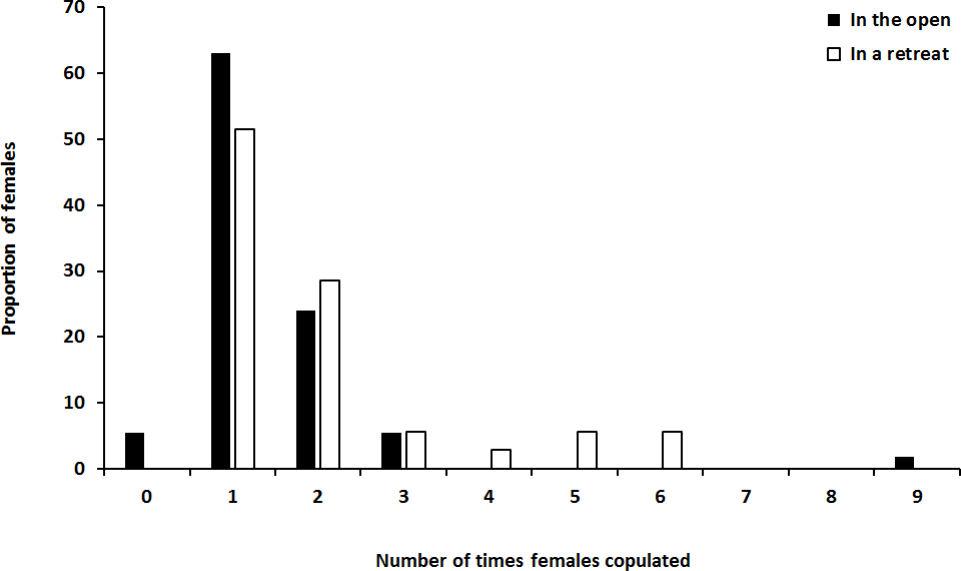
Number of times that female *Servaea incana* mated over a lifetime of repeated exposure to males (a) in the open (*N* = 54) and (b) in a retreat (*N* = 35).

Of the 51 females that had mated as virgins in the open, 17 (33%) remated at least once in their lifetime. Of the 35 females that had mated as virgins in a retreat, 17 (49%) remated at least once in their lifetime (Fig 2). Tendency to remate at least once over a lifetime did not differ significantly between the mating contexts (*G*_1_ = 0.963, *P* = 0.326), with female size (*G*_1_ = 0.488, *P* = 0.485) or with size of the female’s first mate (*G*_1_ = 0.389, *P* = 0.533). Similarly, the number of times that females remated over a lifetime (Fig 3) did not differ between the mating contexts (*G*_1_ = 1.738, *P* = 0.187), with female size (*G*_1_ = 0.252, *P* = 0.616) or with size of the female’s first mate (*G*_1_ = 0.854, *P* = 0.355).

Although lifetime remating tendency did not differ between contexts, amongst those females that did remate at least once the persistence of sexual inhibition did differ. Once-mated females tended to reject fewer males (square root transformed) before remating when tested in the open (Least squares mean ± se = 4.63 ± 0.41, range 0–28) than when tested at a retreat (Least squares mean ± se = 12.13 ± 0.41, range 0–32) (*F*_1,24_ = 4.918, *P* = 0.036). Number of males rejected by females before remating did not vary significantly with size of female (*F*_1,24_ = 0.009, *P* = 0.926) or the size of her first mate (*F*_1,24_ = 0.846, *P* = 0.367).

### Experiment 2: Timing of sexual inhibition onset

All female spiders tested in the open and at the retreat mated with the first male that they encountered in this experiment. Sexual inhibition of females was evident immediately following the female’s first mating in both contexts. Of the 22 virgin females that mated in the open, none remated with the male offered immediately after their first mating (Fisher’s exact test comparing mating tendency when virgin *vs*. when once-mated, *P* < 0.001) and only one remated three hours later giving an overall count of 1 female (4.5%) remating when provided two further opportunities on the day of their first mating. Of the 22 virgin females that mated in retreats, three remated with the male offered immediately after their first mating (Fisher’s exact test comparing mating tendency when virgin *vs*. when once-mated, *P* < 0.001) and none remated three hours later giving an overall count of 3 females (13.6%) remating when provided two further opportunities on the day of their first mating.

## Discussion

### Lifetime mating tendency

Reduced sexual receptivity after mating has been reported in females of many spiders but receptivity patterns over the entire lifespan have not been reported previously. Such information is an important step toward understanding the evolutionary significance of mating-induced sexual inhibition in spiders. In the present study we report the number of times that female *S*. *incana* jumping spiders mated over a lifetime, and the occurrence and latency of sexual inhibition. Female *S*. *incana* were mostly unreceptive immediately after their first copulation and, for many, this mating-induced sexual inhibition persisted for the entire life of the spider (Figs 2 and 3). Most females mated only once or twice in their lifetime.

Mating-induced sexual inhibition in females may reflect the interests of either sex, but is most often considered from the male perspective, perhaps because the benefits for males are easier to identify [2]. For males at risk of sperm competition, an ability to induce sexual inhibition in mates is expected to increase the effectiveness with which copulations convert to fertilizations [11]. Moreover, because sexual inhibition is already induced at the end of the first mating in *S*. *incana*, there is little need for males to invest in mate guarding to prevent matings by rival males. Rather than investing in defence of a transferred ejaculate, it appears that males can accrue higher fitness returns by leaving immediately after mating to search for additional mating opportunities. In the field, *S*. *incana* males can be found wandering on the trunks of trees and females are likely to encounter other males soon after their first mating [80]. In *Gastheracantha minax* orb-weaving spiders, mating-induced sexual inhibition is only expressed in females after 1 to 24 hours and males guard females during this post-mating period of receptivity [86]. Through rapid induction of sexual inhibition in females, *S*. *incana* males appear to avoid this expense.

For *S*. *incana* males, virgin females appear to be a more valuable resource than mated females; virgin females are more likely to mate and do not contain sperm from rival males. Under these conditions, male strategies designed to maximise access to virgin females are anticipated. In nature, as in other spider families [87,88,89], male jumping spiders are commonly found cohabiting with immature females that just need one moult to become adult (‘subadult females’) [90,91,39,64]. After as much as several weeks of cohabitation, these males mate when the subadult female matures [91]. The substantial amount of time that males dedicate to cohabiting provides an indication of the fitness premium that male jumping spiders place on virgin females. Further, a distinct pattern of protandry has been found in field populations of *S*. *incana* [92]; both sexes have a seasonal peak of maturation over a period of 3–4 weeks with male maturation preceding female maturation by several weeks. This is consistent with selection on males to mature at a time that maximises access to virgin females.

In addition to benefits for males, mating-induced sexual inhibition can also be driven by benefits to females. Differences in mating tendency of virgin and mated females may represent changes in mating preferences of females that relate to reproductive security. While it might be important for females to have sperm from the best possible mate to fertilize her eggs, it is also important that females balance this benefit against the risk of failing to mate at all if they are overly discriminating or insufficiently receptive [77]. A first mate is necessary for reproductive security, and so high receptivity levels of *S*. *incana* virgins might be interpreted as relaxation of mate preference criteria to ensure that females accept at least one mate. Such relaxation of criteria may be through a general lowering of thresholds for acceptance or may be through a less strict application of the same criteria used once mated. This possibility has been raised previously by Jackson [77] in reference to another jumping spider, *P*. *johnsoni* that also exhibits high levels of mating-induced sexual inhibition. Once mated, however, females are released from the risk of outright reproductive failure and can turn their attention to selecting high quality mates [93]. If this is the case then we anticipate that those females that never remated tended to be those that had mated with a preferred male type when virgin, whereas those that did remate, and especially those that remated early, were those that would benefit from an ‘upgrade’.

It is unclear at this time whether mating-induced sexual inhibition of jumping spiders represents a general sexual inhibition that applies regardless of male quality or instead represents a sharp increase in mate discrimination such that most males can mate with a virgin but only high quality males can mate with previously mated females. Despite the advantages that large *S*. *incana* males have in terms of performance capacity [94] and contests [95,96], we found no evidence to suggest that mating-induced sexual inhibition was affected by size of a female’s first mate in the present study. At this time, male traits associated with female preferences are poorly understood in jumping spiders.

### Context- and size-dependent mating behaviour

Matings inside retreats entailed longer periods of mounting than was observed for matings in the open. Inside the retreat the copulating pair is more protected and this might have influenced willingness of both the male and the female to remain mounted for longer. Longer matings in the dark or in retreats have been reported for virgin females of other jumping spiders, including *Phidippus johnsoni* [38], *Myrmarachne lupata* [90] and *Trite planiceps* [39]. At this time, it is unclear to what extent mating duration in *S*. *incana* is an expression of male and female influences.

We did not find any evidence of links between female remating tendency and context of copulation, female size or size of the female’s first mate. However, we did find that virgin females in the open rejected more males before their first mating than was the case for females in retreats. Copulating in the open may be associated with a higher risk of predation [97,38,39] and this likely explains why females are less receptive to mating, or more discriminating, in this context.

In the open, larger females tended to reject fewer males before mating, a tendency that was not found in trials at the retreat. In salticids, encounters that occur in the open are more dependent on visual communication than are encounters that occur inside retreats [98]. Males of *S*. *incana* might more readily obtain information about the size of the females in the open. Large size is commonly associated with higher fecundity in female spiders and males of some other spider species appear to show a preference for mating with larger and more fecund females [Metidae, 46; Salticidae, 64,72; Araneidae, 99]. If males of *S*. *incana* prefer larger females, then they might court large females more vigorously or persistently, and gain greater success as a consequence. In *Nephila clavipes* males preferentially cohabit with large females [100] and they also display more vigorously when courting large females [101].

The mechanisms responsible for mating-induced sexual inhibition in females of *S*. *incana* are unknown, but as with insects and ticks physical stimulus during copulation, sperm, and substances in the seminal fluid transferred with the sperm during copulation might be involved [for reviews, see 21,7,12,102]. In *Schizocosa malitiosa* wolf spiders, females copulated by males that are unable to transfer ejaculate maintain high receptivity and this suggests that physical stimulation of mounting and copulation are insufficient to trigger sexual inhibition [42]. Given that other stimuli during copulation were preserved in that study, the authors suggested that, as in many insects, substances in the seminal fluids are responsible for mating-induced sexual inhibition. Two studies provide some insight into the substances that might be responsible of inducing remating inhibition in spiders. Proteinaceous secretions have been found in the male genital tract of the spider *Pholcus phalangoides* and it has been suggested that these secretions are transferred as part of the ejaculate and then act on female physiology and receptivity after mating [103]. Also, Michalik et al. [104] found secretory vesicles in the seminal fluid of the wolf spider *Schizocosa malitiosa*, and hypothesised that these seminal secretions could be responsible for the inhibition of female receptivity. However, the function of such secretions remains to be investigated in spiders.

### Conclusions

The present study is the first to report lifetime mating and remating tendencies of females in a jumping spider and also highlights the involvement of mating context and body size as influences on female mating tendency. Mating-induced sexual inhibition is expressed immediately at the end of the female’s first copulation, and more than half of the tested females rejected all subsequent suitors for life. Mating-induced sexual inhibition plays a huge role in the mating systems and reproductive strategies of many spider taxa and yet much remains to be learned about mechanisms and evolutionary implications.
